# Where is the supporting evidence for treating mild to moderate chronic obstructive pulmonary disease exacerbations with antibiotics? A systematic review

**DOI:** 10.1186/1741-7015-6-28

**Published:** 2008-10-10

**Authors:** Milo A Puhan, Daniela Vollenweider, Johann Steurer, Patrick M Bossuyt, Gerben ter Riet

**Affiliations:** 1 Horten Centre for Patient-oriented Research and Knowledge Transfer, University of Zurich, Switzerland; 2 Johns Hopkins Bloomberg School of Public Health, Department of Epidemiology, Johns Hopkins University, Baltimore (MD), USA; 3 Academic Medical Center, Department of Clinical Epidemiology, Bioinformatics and Biostatistics, University of Amsterdam, 1105 AZ Amsterdam, The Netherlands; 4 Academic Medical Center, Department of General Practice, University of Amsterdam, 1105 AZ Amsterdam, The Netherlands

## Abstract

**Background:**

Randomised trials comparing different drugs head-to-head are extremely valuable for clinical decision-making. However, it is scientifically and ethically sensible to demand strong evidence that a drug is effective by showing superiority over a placebo before embarking on head-to-head comparisons of potentially ineffective drugs. Our aim was to study the evolvement of evidence from placebo-controlled and head-to-head trials on the effects of antibiotics for the treatment of mild to moderate exacerbations of chronic obstructive pulmonary disease.

**Methods:**

We conducted a historical systematic review. Through electronic databases and hand-searches, we identified placebo-controlled and head-to-head antibiotic trials for the treatment of mild to moderate chronic obstructive pulmonary disease exacerbations. We compared the numbers of patients recruited in placebo-controlled and head-to-head trials between 1957 and 2005. Using cumulative meta-analysis of placebo-controlled trials, we determined when, if ever, placebo-controlled trials had shown convincing evidence that antibiotics are effective in preventing treatment failure in patients with mild to moderate chronic obstructive pulmonary disease exacerbations.

**Results:**

The first head-to-head trial was published in 1963. It was followed by another 100 trials comparing different antibiotics in a total of 34,029 patients with mild to moderate chronic obstructive pulmonary disease exacerbations. Over time, the cumulative odds ratio in placebo-controlled trials remained inconclusive throughout with odds ratios ranging from 0.39 (95% confidence intervals 0.04–4.22) to the most recent estimate (1995) of 0.81 (95% confidence intervals 0.52–1.28, *P *= 0.37).

**Conclusion:**

Placebo-controlled trials do not support the use of antibiotics in chronic obstructive pulmonary disease patients with mild to moderate exacerbations. Conducting head-to-head trials is, therefore, scientifically and ethically questionable. This underscores the requirement to perform or study systematic reviews of placebo-controlled trials before conducting head-to-head trials.

## Background

The Helsinki Declaration emphasises the great importance of conducting experimental studies for medical progress. However, it also states that one should be very careful before embarking on randomised trials with placebo controls because research participants have a right to the best available treatment [1]. Worries about the 'unethical use of placebo' continue [2,3]. However, what about the reverse scenario? Might there be cases where experimental treatment did not show superiority over placebo but where the placebo controls were abandoned nevertheless, thus exposing patients to adverse effects and society to healthcare expenditures not offset by any beneficial effects?

There is a large number randomised trials comparing different antibiotics (without placebo control) for the treatment of exacerbations of chronic obstructive pulmonary disease (COPD). Although it may seem plausible that antibiotics are beneficial in about 50% of the COPD patients in whom bacteria are the cause of the exacerbation [4], there is evidence indicating that antibiotics have a short-term effect only in COPD patients with severe exacerbations but not in mild to moderate exacerbations [5]. Although head-to-head comparisons of different treatment options available in clinical practice can be very useful [6], an underlying assumption of such trials is that the treatments are effective compared with a placebo [7-9]. While conducting a series of systematic reviews of treatments in COPD, we gained the impression that the evaluation of antibiotics for COPD exacerbations had moved to head-to-head comparisons of different antibiotics with very little research in placebo-controlled trials. We set out to test this hypothesis in a more systematic way. We counted the number of randomised trials of antibiotic treatment for mild to moderate exacerbations in COPD patients published in the last 50 years, determined whether they used a placebo control or conducted head-to-head comparisons, and contrasted the number of patients studied with the results of a cumulative meta-analysis, using data from a recently published systematic review.

## Methods

### Data sources and searches

For the identification of antibiotics trials in COPD patients, we used a comprehensive literature search described elsewhere [5]. In brief, information specialists (Bazian, London, UK, ) searched the Cochrane Central Register of Controlled Trials (CENTRAL, 2005 issue 4), PREMEDLINE (1960 to 1965), MEDLINE (1966 to March 2006), EMBASE (1974 to March 2006), the Database of Abstracts of Reviews of Effects (DARE, March 2006) for any randomised trials on antibiotics in COPD patients. Two reviewers independently assessed the titles and abstracts of all the identified citations without imposing any language restrictions. The full text of any article that seemed potentially eligible by one of the reviewers was ordered. The reviewers then evaluated the full text of retrieved articles and selected those meeting the inclusion criteria. Each reviewer's decisions were recorded in the Endnote file and any disagreements were resolved by consensus.

### Study selection

We included randomised head-to-head trials comparing any antibiotics or an antibiotic with placebo for the treatment of mild to moderate exacerbations in COPD patients. Placebo-controlled trials allow for an estimation of the specific effects of the treatment and offer a proof of effectiveness [7].

As with any systematic review on this topic, a difficulty is that definitions of COPD have varied over time. In particular, spirometry criteria became widespread only after 1995 and we accepted a clinical diagnosis of COPD, chronic bronchitis or emphysema. However, in order to include only trials whose patients were very likely to have COPD, we included studies only if patients with chronic bronchitis were at least 40 years of age and/or if at least 80% were smokers or ex-smokers. The presence of these characteristics (chronic bronchitis, age and smoking history) renders a diagnosis of COPD according to modern (spirometric) criteria extremely likely [10]. We defined patients to have 'mild to moderate exacerbations' if they needed any outpatient treatment (level I exacerbation) according to the Operational Classification of Severity of the European Respiratory and American Thoracic Societies [11].

### Data extraction and analysis

For each trial, one reviewer recorded details about the type of trial (placebo-controlled or head-to-head), year of publication and number of included patients. A second reviewer checked data extraction for correctness. We calculated the cumulative number of patients included in these trials over the years. We plotted the cumulative number of patients against the year of publication. A systematic review of the placebo-controlled trials has been reported elsewhere [5]. We conducted a cumulative meta-analysis of the effects of antibiotics on treatment failure, commonly used as the main outcome, using the set of placebo-controlled trials. With the cumulative meta-analysis we determined when, if ever, placebo-controlled trials had shown convincing evidence that antibiotics were effective in preventing treatment failure in mild to moderate exacerbations. Thus the cumulative meta-analysis showed what the state of knowledge would have been if people had reviewed the published literature. In a sensitivity analysis, we included another trial [12] that we, as reported previously [5], excluded from the main analyses because we had major doubts about the reported results. We were interested in whether inclusion of this trial changed the cumulative odds ratio (OR) significantly. We conducted all analyses in STATA for windows version 9.2 (Stata Corp; College Station, TX, US).

## Results

### Placebo-controlled and head-to-head trials

Out of 15 placebo-controlled trials, seven enrolled outpatients with mild to moderate exacerbations. We identified a total of 212 head-to-head trials, of which 101 explicitly enrolled outpatients with mild to moderate exacerbations and 63 enrolled inpatients. In 48 trials, patients with any severity of exacerbations were enrolled or severity of the exacerbation could not be determined.

**Figure 1:**
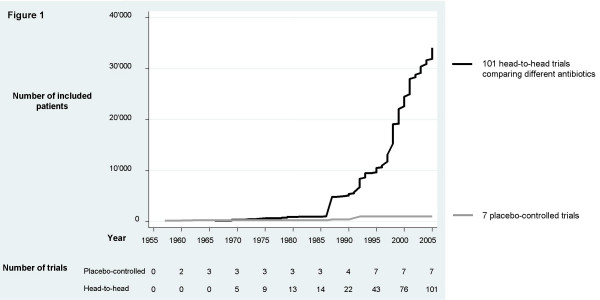
**Number of patients in antibiotic trials**. Cumulative number of patients with chronic obstructive pulmonary disease enrolled in placebo-controlled and head-to-head trials evaluating antibiotics in patients with mild to moderate exacerbations from the first published trials until 2005.

Figure 1 shows the cumulative number of placebo-controlled and head-to-head trials as well as the number of enrolled patients between 1957 and 2005. Over this period of almost 50 years, the 101 head-to-head trials enrolled a total of 34,029 patients with mild to moderate exacerbations.

The first head-to-head trial in 1963 [13] included patients with mild to moderate exacerbations and compared sulphonamide with penicillin, with the aim of finding an antibiotic with fewer adverse events than oxytetracycline, the drug used in the three earlier placebo-controlled trials [14-16]. As in many of the following head-to-head trials [17], the role of the antibiotic itself was not questioned. A common reason to justify a head-to-head trial was that antibiotics are effective against organisms most commonly associated with purulent sputum in chronic bronchitis, such as *Haemophilus influenzae *and *Streptococcus pneumoniae*. Thus rather than citing evidence from placebo-controlled trials, they referred to the *in-vitro *activity of antibiotics (for example [18,19]). Yet another group of head-to-head trials referred to placebo-controlled trials to justify their head-to-head trials but selectively cited only those trials with positive results [20,21].

### Evidence on the effects of antibiotics in mild to moderate COPD exacerbations

**Figure 2:**
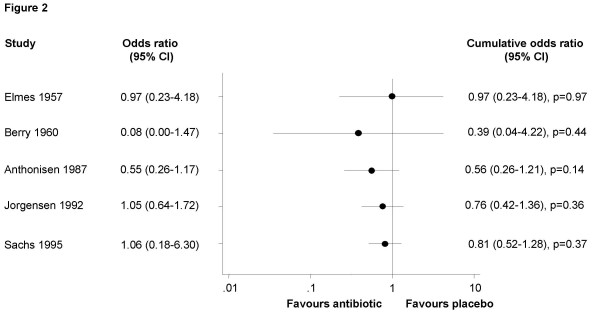
**Evidence on the effects of antibiotics in chronic obstructive pulmonary disease exacerbations**. Cumulative meta-analyses of placebo-controlled trials, which evaluated the effects of antibiotics on treatment failure in patients with mild to moderate exacerbations.

The seven trials included a total of 990 outpatients with mild to moderate exacerbations. Figure 2 shows the cumulative meta-analyses for the five trials reporting on treatment failure. In one trial, treatment failure was defined as event-based because of the need for further antibiotics [15] and in four trials, a symptom-based definition of treatment failure was used [14,22-24]. Cumulative ORs never reached statistical significance and the most recent (1995) cumulative OR was 0.81 (95% confidence interval (CI) 0.52–1.28, *P *= 0.37). When we also included another trial [12], as explained above, the cumulative OR also remained non-significant (0.50, 95% CI 0.20–1.24, *P *= 0.14). In other words, cumulative evidence from placebo-controlled trials did not show any significant effects of antibiotics on treatment failure in COPD patients with mild to moderate exacerbations.

The rationale for the first placebo-controlled trials was reported to be the uncertainty about the benefits of a 'short course' of 'intermittent' antibiotic therapy for exacerbations [14-16]. The authors explicitly stated the short duration of antibiotic treatment because prophylactic long-term use of antibiotics to prevent exacerbations was quite common at that time [25]. Over the following 40 years, authors of placebo-controlled trials argued that these trials are required because 'the role of the [antibiotic] therapy is not clear' [22], or because 'it is still not sufficiently clarified whether acute exacerbations of chronic bronchitis should be treated with antibiotics' [23]. These statements reflect the ongoing debate about the usefulness of antibiotics for COPD exacerbations.

## Discussion

Our historical analysis showed that many head-to-head trials of antibiotics for mild to moderate COPD exacerbations have been conducted although evidence from randomised placebo-controlled trials never showed that antibiotics were effective at all. So, in this case, the evaluation of antibiotics did not follow the general principle that placebo-controlled trials must have shown that the treatment is better than the placebo or a sham procedure, before head-to-head trials are to be conducted [9].

Our study has some strengths and limitations. We used a comprehensive literature search to identify trials. The selection of a widely prescribed drug for a major disease makes our 'case report' meaningful, because we could base our historical analysis on a large number of trials. A limitation of systematic reviews in COPD is the evolution of definitions and classifications of COPD over the years. This raises uncertainty about the nature of the study populations included in trials published before 1995 or even before 2000. Many trials included patients with 'chronic bronchitis' who did not present evidence of chronic airflow obstruction or poor reversibility of airflow obstruction.

It should be emphasised that the results of this study apply to antibiotic treatment for mild to moderate COPD exacerbations. In patients with severe exacerbations, antibiotics show a strong effect not only on treatment failure but also on mortality [5]. Our analysis of placebo-controlled trials differs to some extent to that of the Cochrane review [26] because we did not include a study [27] that was not a randomised but, in our opinion, a matched controlled study. However, we included the trial by Sachs et al [24] missed by the Cochrane review and received the original data from the authors. However, the Cochrane review [26] also showed that antibiotics have a significant effect on treatment failure in hospitalised patients but not in outpatients.

We have focused in our analysis on one major treatment for COPD patients. It remains unclear if head-to-head trials are conducted frequently in other areas without an evidence base from placebo-controlled trials. It is conceivable that this example of antibiotics is just the tip of the iceberg. A recent systematic review summarised placebo-controlled and head-to-head trials of exercise for low back pain [28]. Head-to-head trials were conducted over the same period of time as the placebo-controlled trials. The same applies to trials evaluating antidepressants for elderly people [29,30] or antibiotics for acute otitis media in children [31,32]. For these widely prescribed treatments for common conditions, it remains as yet uncertain whether the conduct of head-to-head trials was scientifically and ethically justified.

Further studies are warranted to estimate the magnitude of the phenomenon that conduct of head-to-head trials is not solidly based on evidence from placebo-controlled trials. It is unclear why investigators rarely refer to existing systematic reviews to justify their trials scientifically and ethically, although access to such systematic reviews has become so easy [33]. We also need to find out what triggers investigators to conduct head-to-head trials too early. The reports on head-to-head trials on antibiotics shed some light on this tendency. High plausibility that an intervention works may be one of the most important reasons. For antibiotics, evidence is available about their *in-vitro *activity against the bacteria commonly found in COPD exacerbations. Intuitively, one may extrapolate this to the clinical situation. Physicians are also confirmed in their habit of antibiotic prescription because most outpatients with mild to moderate exacerbations recover within 2 weeks. Yet they do not seem to be aware of the 'natural' recovery rate (that is, without antibiotics) in these patients, which is 80% or more and equal to that in patients receiving placebo [5].

In general, the pharmaceutical industry tends to favour placebo-controlled trials in order to show large effects and to avoid direct comparison with competitors. In situations like this, however, where comparisons with placebo do not favour a drug, the pharmaceutical industry might be more interested in head-to-head trials. Once a treatment, such as antibiotics for COPD exacerbations, is established in clinical practice an attractive market is available. If a company wants to enter this market it needs to provide a trial showing clinical non-inferiority of a new antibiotic and some advantages in terms of adverse effects or costs. Hence, chance aside, even a new antibiotic lacking specific activity will not be inferior to any established antibiotic in COPD outpatients. This will make it relatively easy to get approval from regulatory agencies, as long as the drug is safe.

As mentioned above, placebo-controlled trials continue to be conducted even if the effectiveness of a drug has been definitively established. Fergusson et al [2] provided an impressive example on that problem recently. They identified all comparisons of aprotinin, which limits perioperative bleeding and reduces the need for blood transfusions, with a placebo. After 12 placebo-controlled trials, the effect estimates stabilised at a cumulative OR of 0.25 for the need for blood transfusions favouring aprotinin. Nevertheless, another 52 randomised placebo-controlled trials were conducted although that was ethically not justifiable [3]. These trials cited previous randomised controlled trials very poorly so that readers were not aware that the question had been answered definitively by previous trials.

The unjustified conduct of placebo-controlled or head-to-head trials can be prevented if funding bodies and ethical committees ask investigators to present a systematic review that justifies a new trial. Such systematic reviews are often available already, for example, published by the Cochrane Collaboration. If not, investigators need to carry out a systematic review themselves and publish it regardless of the findings in order to stimulate a public discussion about the need for new trials. Uncertainty about the effectiveness of a treatment should be discussed openly because it may also encourage patients and physicians to participate in a new trial.

## Conclusion

Our historical analysis showed that the evaluation of antibiotics for mild to moderate COPD exacerbations has been far from optimal. Head-to-head trials have been conducted without any supporting evidence from placebo-controlled trials. This phenomenon raises important scientific and ethical concerns and more studies are warranted to find out its prevalence, mechanisms and consequences in medicine. In the meantime, public funding bodies and ethical committees in particular should encourage the careful conduct or study of pertinent systematic reviews before supporting the conduct of new trials.

## List of abbreviations

CI: confidence interval; COPD: chronic obstructive pulmonary disease; OR: odds ratio

## Authors' contributions

All the authors conceived the study idea. MAP, PB and GT designed the study; MAP and DV collected the data; MP and GT analysed the data. All the authors revised the manuscript and approved the final version of the submitted publication.

## Pre-publication history

The pre-publication history for this paper can be accessed here:


